# Early effects of cigarette smoke extract on human oral keratinocytes and carcinogenesis in head and neck squamous cell carcinoma

**DOI:** 10.1002/hed.26247

**Published:** 2020-05-21

**Authors:** Elisabeth Foki, Katharina Gangl, Veronika Kranebitter, Verena Niederberger‐Leppin, Julia Eckl‐Dorna, Robert Wiebringhaus, Dietmar Thurnher, Gregor Heiduschka

**Affiliations:** ^1^ Department of Otorhinolaryngology, Head and Neck Surgery Medical University of Vienna Vienna Austria; ^2^ Clinical Pharmaology Medical University of Vienna Vienna Austria; ^3^ Clinical Pathology Medical University of Vienna Vienna Austria

**Keywords:** cigarette smoke extract, early carcinogenesis, leukoplakia, oral squamous cell carcinoma, smoking

## Abstract

**Background:**

Cigarette smoking is a major risk factor for head and neck squamous cell carcinoma. Still, the effect of cigarette smoke on the molecular level is unclear. The aim of the present study was to investigate the early effects of cigarette smoke on carcinogenesis of head and neck squamous cell carcinoma.

**Methods:**

Human oral keratinocytes were exposed for 1 week to standardized cigarette smoke extract, and subsequently RT‐quantitative PCR array was performed. Protein expression of dysregulated genes was determined by immunohistochemistry in tissue samples of oral squamous cell carcinoma, oral leukoplakia, and tonsil mucosa.

**Results:**

RT‐PCR revealed upregulation of ITGA‐2 and MMP‐1, whereas TEK receptor tyrosine kinase was downregulated in human oral keratinocytes. ITGA‐2 and MMP‐1 were significantly overexpressed in tissue samples of oral squamous cell carcinoma in comparison to normal mucosa (*P* <.01 in all experiments).

**Conclusion:**

Upregulation of ITGA‐2 and MMP‐1 induced by cigarette smoke contributes significantly to oral carcinogenesis.

## 1 INTRODUCTION

Accounting for more than 650 000 new cases annually,^1^ squamous cell carcinoma of the head and neck (HNSCC) represents the sixth most common cancer in the world. Major risk factors for this type of cancer include human papilloma virus, alcohol abuse, and nicotine.^1^, ^2^, ^3^, ^4^

Worldwide, more than a billion people smoke cigarettes on a daily base.^5^ Clinically, it is well established that cigarette smoke causes various types of malignancies, like cancer of the bladder,^6^ gastric cancer,^7^ cancer of the upper^8^ and lower airway,^9^ and cancer of the oral cavity.^4^, ^8^ The risk for HNSCC is 10 times higher in smokers than in nonsmokers.^4^

However, the influence of cigarette smoke on specific cells is not very well understood. Cigarette smoke is a very heterogeneous mixture of chemical compounds containing more than 3800 substances, including high concentrations of free radicals forming further reactive compounds.^10^, ^11^ There is a large body of evidence about the effects of smoking on human lung epithelium. The most well‐known alterations are changes in p53‐gene and KRAS expression.^8^, ^12^

In contrast, only a few studies report on the effects of cigarette smoke in oral mucosa.^13^ Clinically, cigarette smoke results in oral mucosa dysplasia, leukoplakia, and finally carcinoma.^14^ On the molecular level, it is known that cigarette smoke causes carcinogenesis by inducing severe damage to both somatic DNA and mitochondrial DNA.^15^ In vitro studies showed that these changes result in various posttranslational modifications, for example, upregulation of MMP‐2, AP‐1, and VEGF,^16^.^17^

However, there is little information about how cigarette smoke induces early cellular transformation that leads to the development of malignant tumors in humans.

In this translational study, we investigate the early effects of cigarette smoke on nonimmortalized oral keratinocytes. Furthermore, we assess whether these findings can be observed in oral tissue. We, therefore, investigate tissue samples of healthy oral mucosa, dysplasia, and HNSCC of the oral cavity.

## 2 MATERIALS AND METHODS

### 2.1 Culture of human oral keratinocytes

The primary human oral keratinocyte (HOK) cell line was obtained from ScienCell (SanDiego, California). Cell culturing of HOKs was carried out according to the manufacturer's instructions.

Preparation of cigarette smoke extract was carried out as previously described.^18^, ^19^ In brief, a cigarette smoking machine was used for the production of aqueous cigarette smoke extract (CSE). Two commercially available cigarettes (Marlboro; Philip Morris International Inc., New York, New York; nicotine: 0.8 mg; tar 10 mg) were machine‐smoked at a rate of 15 mL/s for 2 seconds followed by a pause of 28 seconds, cloning the average smoking habits.^20^ CSE was produced by routing the smoke through 8 mL of minimal essential medium (Gibco) containing 100 U/mL penicillin (Gibco), 100 mg/mL streptomycin (Gibco), and 2 mM glutamine (Gibco). The CSE produced in this setting have been standardized to contain a nicotine concentration of 44 ng/mL, a figure comparable to the plasma nicotine concentrations of smokers.^14^

Thus, the dilution used in this study (12.5% of the CSE produced in the machine) equals 5.5 ng nicotine/mL.

### 2.2 RNA isolation and real‐time quantitative PCR array

Human Cancer PathwayFinder PCR Array was obtained from SABiosciences (Quiagen, Valencia, California) and was used to assess alterations in genes responsible for carcinogenesis. RNA was extracted using the RNeasy Mini Kit (Qiagen, Valencia, California) according to the manufacturer's protocol. cDNA was generated using RevertAid Premium First Strand cDNA Synthesis Kit (Fermentas Life Science, St. Leon‐Rot, Germany). All runs were accompanied by a negative control, which included all reagents except cDNA. Gene expression of 84 genes involved in tumorigenesis (including five house‐keeping genes for normalization) was assessed according to the manufacturer's protocol and analyzed on a CFX96 (Biorad). Only genes demonstrating a 2‐fold or greater change were considered as relevant.

### 2.3 Patient data

Tissue samples from 10 patients with oral leukoplakia and 10 tissue samples of oral squamous cell carcinoma were obtained during diagnostic panendoscopy or excisional biopsy, respectively. Ten tissue samples from patients suffering from chronic tonsillitis were obtained during tonsillectomy to receive healthy tissue samples. All patients were treated at the Department of Otorhinolaryngology, Head and Neck Surgery, Medical University of Vienna, Austria. Tissue samples were reviewed by two investigators (R. W. and E. F.).

### 2.4 Immunohistochemistry

Antibodies of ITGA‐2 (integrin alpha 2), MMP‐1 (collagenase‐1), and TEK receptor tyrosine kinase (TIE‐2) corresponding to above‐shown gene expression were obtained (Sigma Aldrich, Germany). Ideal dilution ratios and retrieval buffer was determined before staining (ITGA‐2:1:100, MMP‐1:1:100, TEK: 1:250). Briefly, tissue sections were deparaffinized with xylene and rehydrated with decreasing alcohol concentrations. After heat‐induced epitope retrieval in a microwave oven (600 W) using citrate buffer (10 mmol/L, pH 6.0), slides were incubated at room temperature with the primary antibody directed against ITGA‐2, TEK, and MMP‐1 for 1 hour. UltraVision LP detection system (Lab Vision Corporation, Fremont, California) was used to detect antibody binding in accordance with the manufacturer's recommendations. Antibody binding sites were colored brownish by adding 3‐3‐diaminobenzidine. Finally, counterstaining of the tissue samples with hematoxylin Gill III (Merck, Darmstadt, Germany) was performed.

All slides were assigned to one of four categories of marker expression: 0 = negative; 1 = weak: staining in <30% of cells; 2 = moderate: staining in 30% to 60% of cells; and 3: strong staining in more than 60% of cells. The average of the core stains was taken to determine the staining intensity.

Positive controls were done in accord with the manufacturer's protocol. Samples were analyzed using an Olympus BH‐2 microscope (Olympus America, Melville, New York).

### 2.5 Statistical analysis

In all experiments in the real‐time quantitative PCR array, data are reported as mean ± SEM in three independent replicates. Data were analyzed by post hoc comparisons using two‐tailed *t* test.

Expression profiles of ITGA‐2, MMP‐1, and TEK were compared using *t* test. *P* values of <.05 were considered as statistically significant.

## 3 RESULTS

### 3.1 Alteration of gene expression after exposure to CSE

**FIGURE 1:**
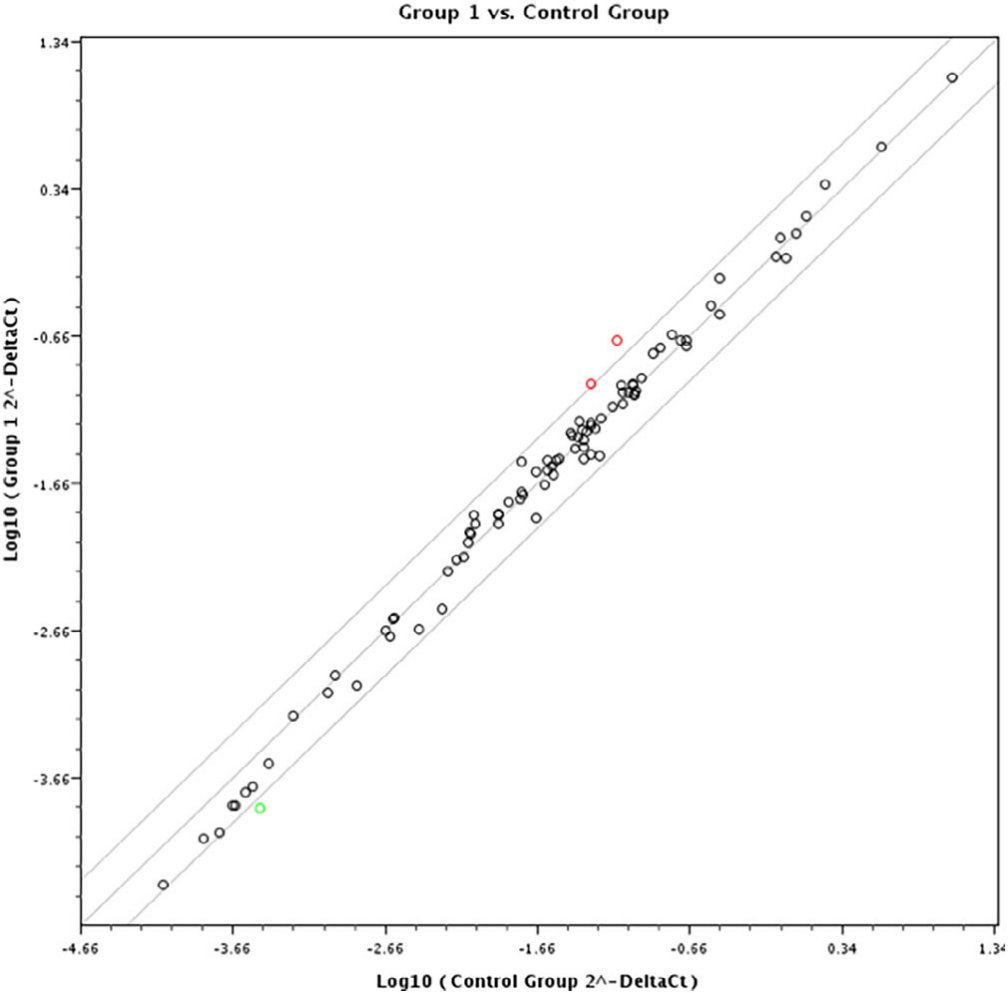
Scatter blot of 84 target genes involved in carcinogenesis of human oral keratinocytes after exposure to cigarette smoke extract. A modulation of two times or more was considered as relevant. The red circles indicate upregulation of ITGA‐2 and MMP‐1, whereas the green circle indicates the downregulated TEK‐gene [Color figure can be viewed at wileyonlinelibrary.com]

To investigate the effect of CSE on HOK at the molecular level, we performed a real‐time PCR array containing 84 genes that are involved in carcinogenesis (Table 1). The relative expression of these target genes was analyzed and the up‐ or downregulation was measured. A change of more than 2‐fold was considered as relevant. Most interestingly, MMP‐1 and ITGA‐2 were upregulated, whereas TEK was downregulated in our cells (Figure 1).

**TABLE 1:** Expression of multiple cancer target genes, including growth factors and receptors investigated by the Cancer PathwayFinder RT^2^ Profiler PCR Array

AKT1	ERBB2	MDM2	SERPINB5 (maspin)
ANGPT1 (angiopoietin‐1)	ETS2	MET	SERPINE1 (PAI1)
ANGPT2 (angiopoietin‐2)	FAS	MMP1 (collagenase 1)	SNCG
APAF1	FGFR2	MMP2 (gelatinase A)	SYK
ATM	FOS	MMP9 (gelatinase B)	TEK (TIE‐2)
BAD	GZMA	MTA1	TERT (telomerase)
BAX	HTATIP2	MTA2	TGFB1
BCL2	IFNA1 (IFNα)	MTSS1	TGFBR1 (ALK‐5)
BCL2L1 (bcl‐X)	IFNB1 (IFNβ)	MYC	THBS1 (thrombospondin‐1)
BRCA1	IGF1	NFKB1 (NFκB)	TIMP1
CASP8	IL8	NFKBIA (IκBα)	TIMP3
CCNE1 (cyclin E1)	ITGA1 (integrin α1)	NME1	TNF
CDC25A	ITGA2 (integrin α2)	NME4	TNFRSF10B (DR5)
CDK2	ITGA3 (integrin α3)	PDGFA	TNFRSF1A (TNF‐a receptor)
CDK4	ITGA4 (integrin α4)	PDGFB	TNFRSF25 (DR3)
CDKN1A (p21Waf1)	ITGAV (integrin αV)	PIK3R1 (PI3K p85α)	TP53 (p53)
CDKN2A (p16Ink4)	ITGB1 (integrin β1)	PLAU	TWIST1
CFLAR (CASPER)	ITGB3 (integrin β3)	PLAUR	VEGFA
CHEK2 (chk2 / Rad53)	ITGB5 (integrin β5)	PNN	
COL18A1 (endostatin)	JUN	RAF1	
E2F1	MAP2K1 (MEK)	RB1	
EPDR1	MCAM	S100A4	

*Note:* Most notably, ITGA2 and MMP‐1 was upregulated in oral keratinocytes exposed to cigarette smoke extract, whereas TEK was downregulated.

### 3.2 Immunohistochemistry

**FIGURE 2:**
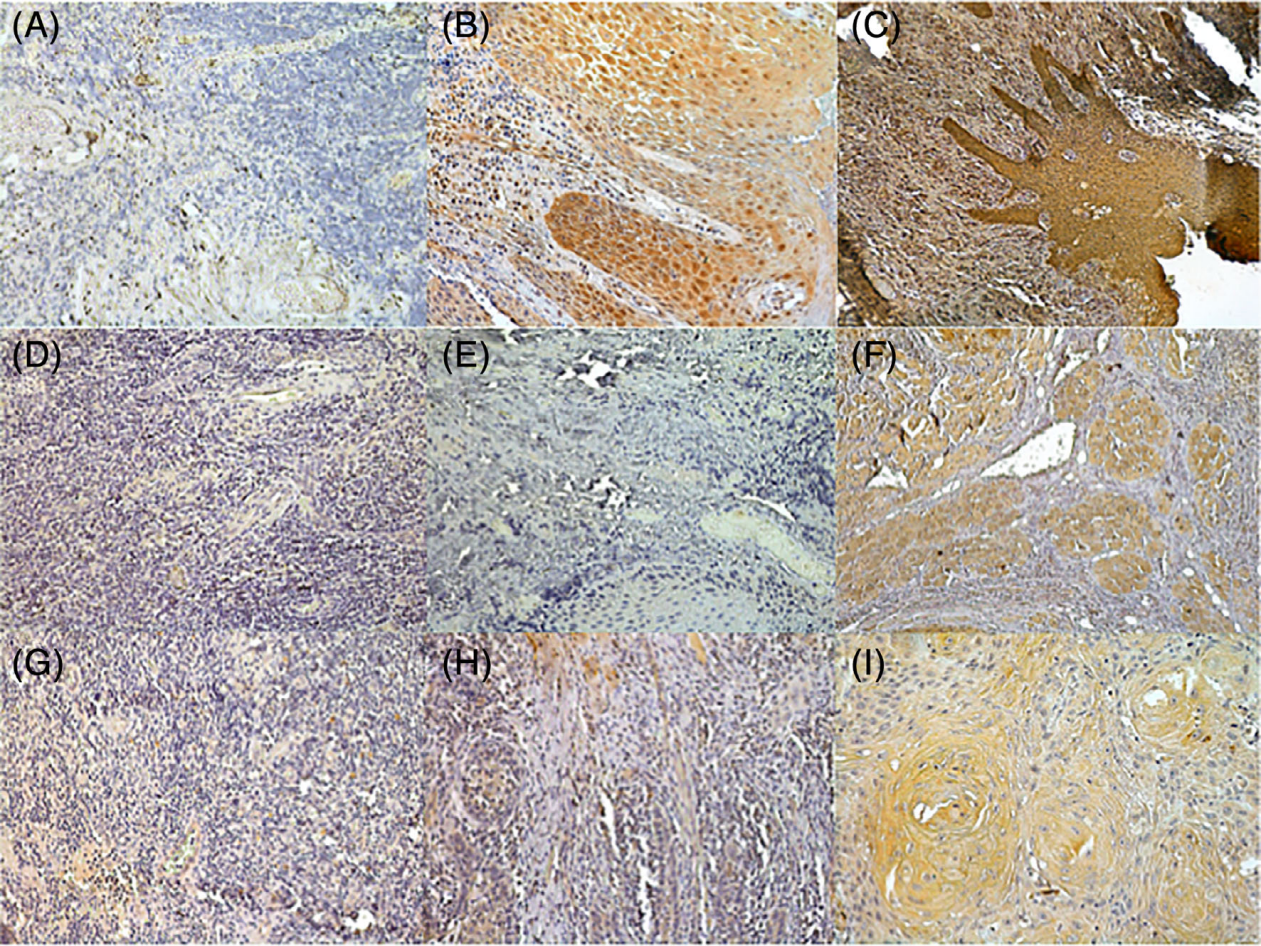
Immunostaining of ITGA‐2 in A, healthy mucosa, B, oral leukoplakia, and C, oral squamous cell carcinoma and of MMP‐1 in D, healthy mucosa, E, oral leukoplakia, and F, oral squamous cell carcinoma, respectively. Immunostaining of TEK in G, healthy mucosa, H, oral leukoplakia, and I, oral squamous cell carcinoma. All photographs were taken at original magnification ×20 [Color figure can be viewed at wileyonlinelibrary.com]

To assess the results from the CSE in vitro experiments in patient material, we stained the respective markers in healthy mucosa, leukoplakia, and oral squamous cell carcinoma (Figure 2, Table 2).

**TABLE 2:** Schematic representation of the results of immunohistochemistry of ITGA‐2, MMP‐1, and TEK in tissue samples of oral squamous cell carcinoma, oral leukoplakia, and oral mucosa

	ITGA‐2	MMP‐1	TEK
Carcinoma	1	3	0
	3	1	1
	3	2	2
	3	3	0
	1	0	0
	2	3	2
	3	3	1
	m	2	1
	3	3	2
	1	1	1
Leukoplakia	2	1	2
	2	2	2
	3	1	m
	3	0	0
	3	1	1
	0	1	2
	2	1	2
	m	0	2
	3	1	1
	3	1	3
Oral mucosa	0	1	0
	0	0	1
	1	1	0
	0	1	0
	2	0	1
	1	1	m
	0	1	1
	2	3	3
	1	0	0
	1	0	1

*Note:* 0 = no expression, 1 = weak expression, 2 = moderate expression and 3 = strong expression. M indicates insufficient staining intensity.

#### 3.2.1 Expression profiles of ITGA‐2

In healthy tonsillar tissue samples, two patients showed moderate expression of ITGA‐2 (20%), four tissue samples showed weak expression of ITGA‐2 (40%), and four tissue samples did not show expression of ITGA‐2 (40%).

Expression profiles in patients with oral leukoplakia was as it follows: five patients showed strong expression (50%), three patients showed moderate expression (30%), one tissue sample did not show expression of ITGA‐2 (10%), and in one tissue sample staining was insufficient (10%).

ITGA‐2 was expressed in oral squamous cell carcinoma as it follows: five patients showed strong expression of ITGA‐2 (50%), one patient showed moderate expression (10%), three patients showed weak expression (30%), and in one tissue sample (10%), staining of ITGA‐2 was insufficient.

#### 3.2.2 Expression profiles of MMP‐1

MMP‐1 expression in tonsillar mucosa was as it follows: one patient showed strong expression (10%), five tissue samples showed weak expression (50%), and four tissue samples (40%) showed no expression of MMP‐1.

MMP‐1 was expressed in oral leukoplakia as it follows: one tissue sample showed moderate expression (10%), seven patients showed weak expression (70%), and two samples showed no expression of MMP‐1 (20%).

Oral squamous cell carcinoma tissue samples showed MMP‐1 expression as it follows: five tissue samples (50%) showed strong expression of MMP‐1, two patients moderate (20%), and two patients weak expression (20%) of MMP‐1. One tissue sample showed no expression of MMP‐1 (10%).

#### 3.2.3 Expression profiles of TEK

TEK was expressed as it follows: Healthy tissue: TEK was strongly expressed in one tissue sample (10%), weakly expressed in four tissue samples (40%). Four tissue samples showed no expression (40%) and staining was insufficient in one tissue sample (10%).

Oral leukoplakia: One patient showed strong expression (10%), five patients showed moderate expression (50%), two patients showed weak expression (20%), and one patient showed no expression (10%). In one tissue sample, staining was insufficient (10%).

Oral squamous cell carcinoma: three patients showed moderate expression (30%), four patients showed weak expression (40%), and three patients showed no expression of TEK (30%).

### 3.3 Statistical analysis of expression profiles

When compared to healthy mucosa, ITGA‐2 was significantly overexpressed in oral leukoplakia (*P* =.002) as well as in oral squamous cell carcinoma (*P* =.003). MMP‐1 was significantly overexpressed in cancerous tissue in comparison to normal mucosa (*P* =.007). However, there were no differences in leukoplakia vs healthy mucosa (*P* =.749).

TEK expression in healthy tissue did neither significantly differ in comparison to normal mucosa (*P* =.595) nor to leukoplakia (*P* =.057).

## 4 DISCUSSION

Global cancer statistics have shown that one of the major risk factors for head and neck squamous cell carcinoma is nicotine abuse.^1^, ^2^, ^3^ Smokers have a 10‐time higher risk for development of HNSCC than never‐smokers.^4^ Cigarette smoke contains a variety of well‐known carcinoma‐inducing and mutagenic substances such as polycyclic aromatic hydrocarbons, tobacco‐specific nitrosamines, and aldehydes.^12^ While there is an abundance of knowledge available regarding the final result, only little is known about the very early effects of cigarette smoke on oral mucosa.

In this study, we tried to identify altered gene expression in smoke‐exposed oral keratinocytes. Subsequently, we investigated consequent posttranslational changes in expression of specific proteins in further progressed transformation states of oral mucosa.

Initially, we analyzed gene expression of oral keratinocytes after exposure to a defined amount of cigarette smoke and compared it to an unexposed cell population. We therefore used a PCR array for cancer specific genes. We could show an upregulation of ITGA‐2 and MMP‐1, which are markers of dysregulation of cell adhesion and protein‐protein interaction, respectively.^21^, ^22^ So far, one group showed an upregulation of both of these genes in squamous cell carcinoma of the skin.^23^ Boyle et al. revealed alterations of various gene groups in oral mucosa of smokers, such as enrichment of prostaglandin and leukotriene metabolism, which is associated with MMP‐1 upregulation in HNSCC.^24^, ^25^ Furthermore, MMP‐1 was significantly overexpressed in carcinoma of the vocal folds in comparison to leukoplakia and therefore it was associated with disease progression.^26^

We also found a downregulation of TEK in smoke‐exposed cells. It was previously described that TEK is downregulated in laryngeal HNSCC treated with chemotherapy.^27^ Furthermore, reduced cell growth in HNSCC was associated with upregulation of TEK.^28^

Subsequently, we investigated protein expression of these findings in tissue samples of healthy tissue, oral leukoplakia, and oral squamous cell carcinoma.

First, we assessed the expression of the integrin ITGA‐2. Integrins are cell surface receptors that regulate cell adhesion to the extracellular matrix and immunoglobulins. It is known that ITGA‐2, is a highly expressed collagen receptor on normal epithelial cells and platelets.^29^ However, it also plays a crucial role in cancer initiation and the development of metastasis.^22^, ^29^ In particular, the integrin alpha subfamily has been shown to be associated with worse prognosis in multiple types of cancer, including nonsmall‐cell lung carcinoma, colon, and HNSCC.^22^ Recently, e‐cadherin, a protein linked to integrin alpha activity, has been detected to be a marker of early oral carcinogenesis.^30^, ^31^ We were able to demonstrate that ITGA‐2 was upregulated in leukoplakia and also in oral squamous cell carcinoma. To our knowledge, this is the first report of increased ITGA‐2 expression in oral keratinocytes as a result of cigarette smoke exposition as well as increased ITGA‐2 expression in oral SCC.

Secondly, we have evaluated the expression of the matrix‐metalloproteinase transmembrane protein MMP‐1. These proteins are able to degrade extracellular matrix and have been associated with tumor progression and metastasis.^21^ O‐Charoenrat et al. could show, that MMP‐1 was overexpressed in HNSCC in comparison to normal adjacent mucosa and also associated with advanced T‐stage.^32^ Also, various tobacco‐related diseases have been associated with elevated MMP‐1 expression.^33^, ^34^ Interestingly, a cigarette smoke responsive promoter region of MMP‐1 has been identified in airway epithelial cells.^33^ Furthermore, Kalfert et al. showed, that serum levels of MMP‐1 are significantly elevated in smokers and thus might influence etiopathology of HNSCC.^35^ So far, no data about the MMP‐1 expression in OSCC tissue samples and smoking are available and we were able to demonstrate an overexpression in oral squamous cell carcinoma in comparison to normal oral mucosa.

The third gene that was regulated after cigarette smoke exposure was TEK. This is an endothelial receptor‐tyrosine kinase, which is one of the key‐regulators of angiogenesis. It was previously described that TEK counteracts the VEGF‐system in latter stages of angiogenesis and vascular maturation.^36^ However, we could not detect significant changes in protein expression in our tissue samples.

Summarizing this study, we could show the early effects of cigarette smoke exposure on oral keratinocytes. We revealed an upregulation of ITGA‐2, MMP‐1, and a downregulation of TEK in oral keratinocytes. To confirm our results on the posttranslational level, we performed immunohistochemistry of oral mucosa, oral leukoplakia, and oral squamous cell carcinoma. Most interestingly, ITGA‐2 and MMP‐1 were significantly overexpressed in cancerous tissue. Taken together, these changes seem to influence various transformation states of oral mucosa and thus contribute substantially to early oral carcinogenesis after cigarette smoke exposure.
